# Osteoperiostitis in children: proposal for a diagnostic algorithm

**DOI:** 10.1007/s00431-021-04058-3

**Published:** 2021-04-08

**Authors:** Francesco Zulian, Elena Marigo, Francesca Ardenti-Morini, Fabio Vittadello, Monica Zuliani, Chiara Giraudo, Alessandra Meneghel, Giorgia Martini

**Affiliations:** 1 Rheumatology Unit, Department of Woman’s and Child’s Health, University of Padova, Via Giustiniani 3, 35128 Padua, Italy; 2 Pediatric Unit, Sant’Eugenio Hospital, Rome, Italy; 3 Explora—Research and Statistical Analysis, Vigodarzere, Padua, Italy; 4 Department of Medicine—DIMED, Radiology Institute, University of Padova, Padua, Italy

**Keywords:** Periostitis, Osteomyelitis, Chronic non-bacterial osteomyelitis, Goldbloom syndrome, Differential diagnosis, Classification tree

## Abstract

Juvenile osteoperiostites (JOP) are a group of inflammatory bone diseases whose differential diagnosis is often difficult. The main conditions are acute osteomyelitis (AOM), chronic non-bacterial osteomyelitis (CNO) and the Goldbloom syndrome (GS). The study was aimed to develop an algorithm to enable an early diagnosis of JOP. Clinical records of patients with AOM, CNO and GS, followed at our Center over the past 10 years, were reviewed. Twelve additional patients with GS were selected from PubMed/MEDLINE literature search. Data collected included demographics, clinical manifestations, laboratory and instrumental investigations at disease onset. The association between categorical variables was investigated, and the segmentation of patients with different diagnoses was analyzed through a classification tree model (CTREE package) in order to build up a diagnostic algorithm. Ninety-two patients (33 CNO, 44 AOM, 15 GS) entered the study. Among 30 variables considered at onset, nine (age at onset, fever, weight loss, symmetry, focality, functional limitation, anemia, elevated ESR, CRP) resulted statistically significant in differentiating the three clinical entities from each other and were chosen to build up a decisional tree. Three variables, symmetry of bone involvement, presence of fever and age at disease onset, resulted significant to discriminate each of the three diseases from the others. The performance of the diagnostic algorithm was validated by comparing the diagnoses provided by the model with the real diagnoses and showed 85.9% accuracy.

*Conclusion*: We propose a diagnostic algorithm, based on simple clinical data, which can help guide a prompt and appropriate diagnosis of JOP.

**Table:** 

**What is Known:** *• Juvenile osteoperiostitis (JOP) are a group of inflammatory bone diseases followed by various pediatric specialists.* *• The distinction between these conditions is not easy as clinical and laboratory features often overlap.* **What is New:** *• We propose a diagnostic algorithm, based on clinical data of real patients, with high degree accuracy.* *• This instrument can help guide the prompt and appropriate diagnosis of JOP.*

**What is Known:**

*• Juvenile osteoperiostitis (JOP) are a group of inflammatory bone diseases followed by various pediatric specialists.*

*• The distinction between these conditions is not easy as clinical and laboratory features often overlap.*

**What is New:**

*• We propose a diagnostic algorithm, based on clinical data of real patients, with high degree accuracy.*

*• This instrument can help guide the prompt and appropriate diagnosis of JOP.*

## Introduction

Juvenile osteoperiostites (JOP) represent a group of challenging inflammatory conditions whose differential diagnosis in children is often difficult, with consequent delay in starting an appropriate treatment. Once excluded a malignancy, the two main conditions included in JOP are acute osteomyelitis (AOM) and chronic non-bacterial osteomyelitis (CNO) [1, 2]. There is also a rarer and perhaps underdiagnosed entity, the Goldbloom syndrome (GS), which deserves to be considered in this context [3].

The distinction between these conditions is not always immediate as clinical signs and symptoms, laboratory and imaging often overlap, with the risk of incorrect or delayed diagnosis.

AOM is an infectious process, typically bacterial, which generally affects the metaphyses of the long bones. Infection from the bone can extend and involve other tissues such as periosteum, surrounding soft tissues and bone marrow. AOM is characterized by acute onset of fever and bone pain which can lead to functional limitation, such as limping. The most frequent etiological agent of AOM is *Staphylococcus aureus*, followed by group A β-hemolytic streptococcus (SβEGA) and *Haemophilus influenzae* type b (Hib) [1]. Generally, the localization of AOM is monofocal, but, in case of severe septicemic processes or in immunocompromised individuals, the presence of multiple foci has been reported [1].

Chronic non-infectious osteomyelitis (CNO) is an autoinflammatory condition characterized by sterile osteomyelitis. CNO has a wide spectrum of clinical manifestations that may range from simple self-limiting inflammatory lesions (mono- or oligo-focal) to more severe forms such as the chronic recurrent multifocal osteomyelitis (CRMO). Diagnosis is clinical, histological and by exclusion of other bone diseases such as tumors and histiocytosis [2].

Goldbloom syndrome (GS) is a rare and still poorly understood clinical condition in which osteoperiostitis presents with bone pain, fever and weight loss, often associated with dysproteinemia [3]. After the first two cases described in 1966 by Goldbloom, 13 more cases have been reported in the literature from 1984 to date [4–9]. The etiopathogenesis of GS is unknown although it is considered a post-infectious condition. Characteristic is the symmetrical localization of the inflammatory process that most frequently affects the long bones: radius, femur, humerus, ulna and tibia, in decreasing order of frequency [5, 9]. Radiological changes mainly involve the periosteum, although inflammation can affect the bone and sometimes the bone marrow [4, 5, 7]. Peculiar of this syndrome are the prompt response to corticosteroid therapy and the slow normalization of laboratory and radiological picture mostly within 4 months.

The aim of this study was to search for clinical features at disease onset that may rapidly address the clinicians towards appropriate investigations for the correct diagnosis of different forms of JOP.

## Methods

We retrospectively analyzed the clinical characteristics of patients with AOM, CNO and GS followed at our Center over the past 10 years. Since GS is a rarely reported condition, patients with comprehensive dataset were also selected from the literature by PubMed/MEDLINE search. We excluded other possible conditions such as malignancy, scurvy, Garre’s syndrome and voriconazole-induced periostitis because of their chronic course and/or peculiar medical history. Data collected included general characteristics of the patients (gender, age at onset, presence of a possible trigger events, family history of rheumatic disease, associated autoimmune diseases, monophasic or recurrent disease course), clinical manifestations present at onset (fever, site of involvement, local signs of inflammation, symmetry and mono or multi-focal bone pain, concomitant arthritis, functional limitations, weight loss, other systemic symptoms and extra bony manifestations) and laboratory investigations (erythrocyte sedimentation rate (ESR) and C-reactive protein (CRP), white blood cell count (WBC), hemoglobin, α_2_-globulin concentration). ESR and CRP were considered abnormal if greater than 30 mm and 5 mg/l, respectively. WBC, hemoglobin and protein profile were considered to be abnormal if greater or lower than 2 SD values for age, platelets if greater than 400,000/mm^3^. The results of blood culture, radiological investigations (standard X-ray, magnetic resonance imaging (MRI), bone scan) and bone biopsy were also collected. Two independent radiologists (MZ and CG) reviewed the radiological imaging coming from our Institution and also those coming from the peripheral hospitals where some patients have been initially admitted. Patients with incomplete data were excluded by the analysis.

### Statistical analysis

As for each variable, the absolute and percentage distributions of the subjects with AOM, CNO and GS were calculated. Comparisons between the three groups were made through the application of appropriate statistical tests, taking into account the characteristics of the variables considered in the analysis and the sample size. The association between categorical variables was investigated using the Fisher’s exact test, and also in its extended version, the Fisher-Freeman-Halton test, if needed. The segmentation of patients with different diagnoses was also analyzed through the classification tree model (using the Classification Trees: Conditional Inference Tree—CTREE package from R). The aim of this approach was to recursively perform univariate subdivisions (with significance < 0.05) of the dependent variable (final diagnosis) based on the values ​​of a set of covariates previously selected with bivariate analysis. Branching procedure continues until the remaining predictors do not have statistically significant univariate associations. The final tree identifies the parameters that clearly separate the patients, highlighting segments with significantly high percentages for each diagnosis. A *p* value < 0.05 was considered statistically significant. All analyses were performed by using SPSS (Vers. 18.0) and R (Vers. 3.6.1) statistical software.

## Results

Ninety-two patients with complete clinical documentation entered the study. Thirty-three had CNO, 44 OMA and 15 GS. As for GS, three patients have been diagnosed at our Center, and twelve additional patients were selected from the literature by PubMed/MEDLINE search. We made this choice because of the rarity of this disease and to make the GS group statistically comparable with the other two.

The clinical characteristics of the patients are summarized in Table 1. The average age at onset was significantly higher in patients with CNO (9 years) in comparison with GS and AOM (6 years). Gender appeared equally distributed in the three groups, while a trigger event, usually a respiratory infection, was identified in 80% of patients with GS. A monophasic course significantly characterized GS and OMA as compared to CNO that presented a recurring course in more than 75% of cases. Fever, globally present at onset in 62% of the patients, was mainly found in GS and AOM. The localization of bone pain in the upper limbs was more frequently present in GS and CNO, rarely in AOM. Conversely, the localization of pain at the lower limbs was prevalent in AOM and GS. The disease was mainly multifocal in GS (93.3%) and CNO (57.6%), monofocal in AOM (95.5%). Symmetrical bone involvement at onset was typical of GS (93.3%), rare in the other two diseases. Therefore, focality, symmetry and associated functional limitation showed good significance in differentiating the three forms from each other. As for systemic symptoms, weight loss was significantly more frequent in GS, having been reported in half of the patients.

**Table 1:** Clinical characteristics of the patients

			CNO *n* 33 (%)	GS *n* 15 (%)	AOM *n* 44 (%)	*p*
Demographics	Age at onset years (range)		9.1 (2–18)	6.2 (0.3–14)	6.1 (0.4–14)	0.000
	Gender	Female	22 (66.7)	8 (53.3)	17 (38.6)	n.s.
	Trigger event	Trauma	6 (18.2)	0 (0%)	8 (18.2)	0.000
		Infection	0 (0)	12 (80)	9 (20.5)	
	Positive family history		8 (24.2)	0 (0)	4 (9.1)	n.s.
Signs and symptoms	Fever		7 (21.2)	14 (93.3)	36 (81.8)	0.000
	Bone pain		29 (87.9)	15 (100)	43 (97.7)	n.s.
	Localization of bone pain	Upper limbs	15 (45.5)	10 (66.7)	5 (11.4)	0.000
		Lower limbs	20 (60.6)	14 (93.3)	37 (84.1)	0.014
		Axial	11 (33.3)	4 (26.7)	3 (6.8)	0.011
	Focality	Monofocal	14 (42.4)	1 (6.7)	42 (95.5)	0.000
		Multifocal	19 (57.6)	14 (93.3)	2 (4.5)	
	Symmetry		4 (12.1)	14 (93.3)	0 (0)	0.000
	Functional limitation		11 (33.3)	6 (40)	31 (70.5)	0.003
	Concomitant arthritis		4 (12.1)	0 (0)	11 (25)	n.s.
	Weight loss		2 (6.1)	8 (53.3)	3 (6.8)	0.000
Course	Clinical course	Monophasic	8 (24.2)	14 (93.3)	42 (95.5)	0.000
		Recurrent	25 (75.8)	1 (6.7)	2 (4.5)	

Values are number (%)

Among laboratory tests, elevation of acute phase reactants, ESR and CRP, neutrophilic leukocytosis, high platelet count and anemia were significantly more frequent in patients with GS and AOM (Table 2). Abnormal protein profile was found in all patients with GS and consisted of hypoalbuminemia and increased α2- and/or γ-globulins. Standard X-ray at onset was abnormal in 93.3% of patients with GS, in 87.9% of CNO but only in 40.9% in AOM. Radiological signs of periostitis characterized GS (80%), while osteitis was prevalent in AOM (31.8%) (Table 2). MRI was pathological in almost all patients, with no significant difference between the three conditions. Bone scan, performed in 70% of patients, confirmed the focal involvement already evident clinically in most of the cases. Bone biopsy, performed in one-third of the patients, was generally nonspecific, with little ability to differentiate between the three conditions. In conclusion, MRI, bone scan and biopsy are of little benefit for the differential diagnosis of JOP but help rule out possible malignancy.

**Table 2:** Laboratory and imaging results at disease onset

			CNO *n* 33 (%)	GS *n* 15 (%)	AOM *n* 44 (%)	*p*
Laboratory	ESR (> 30 mm/h)		15 (45.5)	14 (93.3)	38 (86.4)	0.000
	CRP (> 5 mg/l)		17 (51.5)	15 (100)	37 (84.1)	0.000
	WBC elevation		1 (3)	4 (26.7)	13 (29.5)	0.011
	Anemia		1 (3)	12 (80)	17 (38.6)	0.000
	Thrombocytosis		6 (18.2)	13 (86.7)	17 (38.6)	0.000
	Abnormal protein profile		9 (27.3)	15 (100)	21 (47.7)	0.000
	Positive blood colture		0/12 (0)	0/8 (0)	13/43 (30.2)	0.021
Imaging	Standard X-ray	Abnormal	29 (87.9)	14 (93.3)	18 (40.9)	0.000
		Osteoperiostitis	17 (51.5)	2 (13.3)	4 (9.1)	
		Periostitis	0 (0)	12 (80.0)	0 (0)	
		Osteitis	12 (36.4)	0 (0)	14 (31.8)	
	MRI	Osteoperiostitis	5 (15.6)	3 (42.9)	9 (31)	n.s.
		Periostitis	0 (0)	1 (14.3)	0 (0)	
		Osteitis	27 (84.4)	2 (28.5)	20 (69)	
	Bone scan	Monofocal	9 (27.3)	0 (0)	20 (87)	0.000
		Multifocal	24 (72.7)	8 (100)	3 (13)	

Values are number (%)

### Construction of a decision-making model

**Fig. 1:**
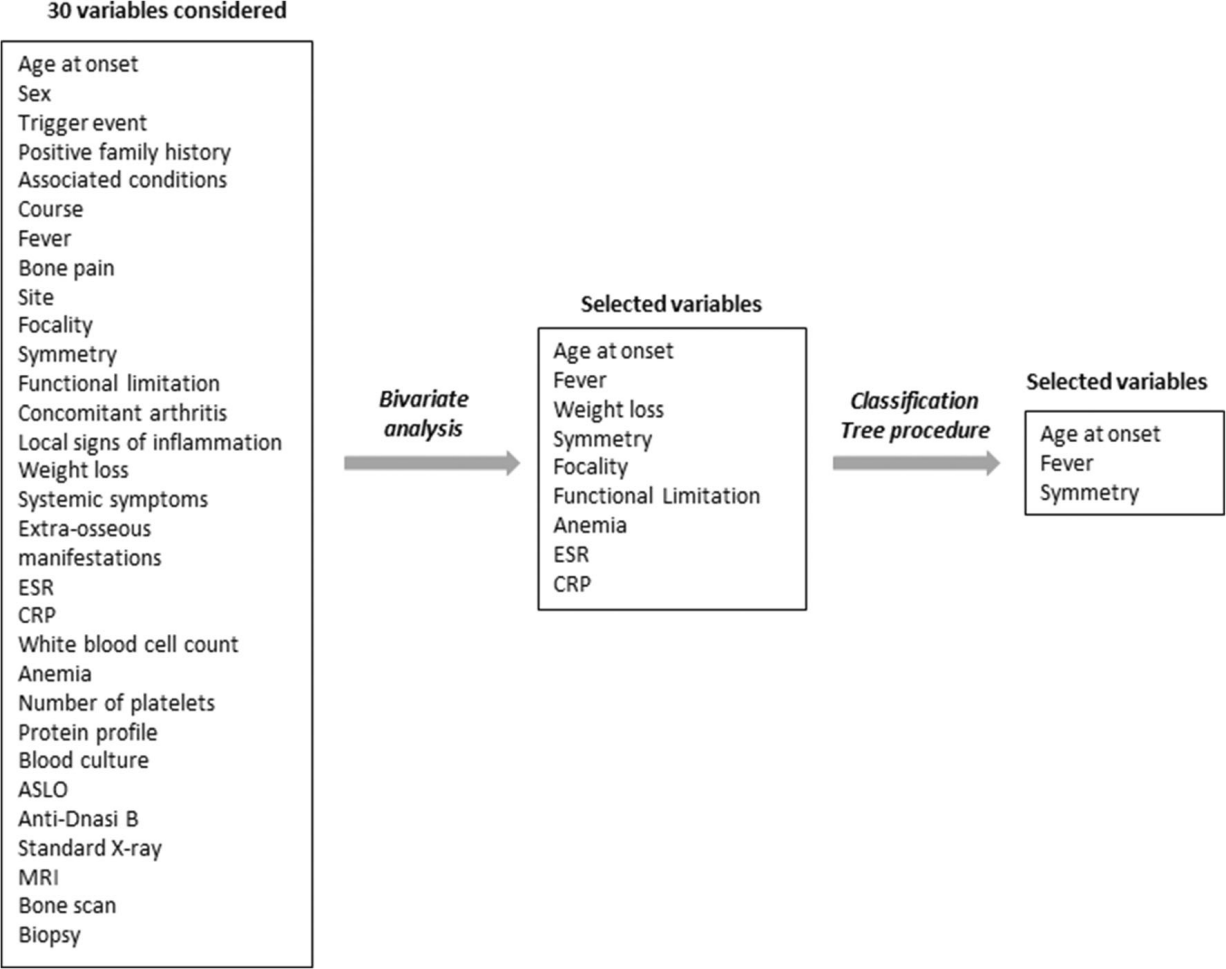
Construction of the decision-making model. From the initial 30 variables, after the bivariate analysis, a set of 9 covariates entered into the R’s Classification Tree procedure that selected the final three decisional nodes

**Fig. 2:**
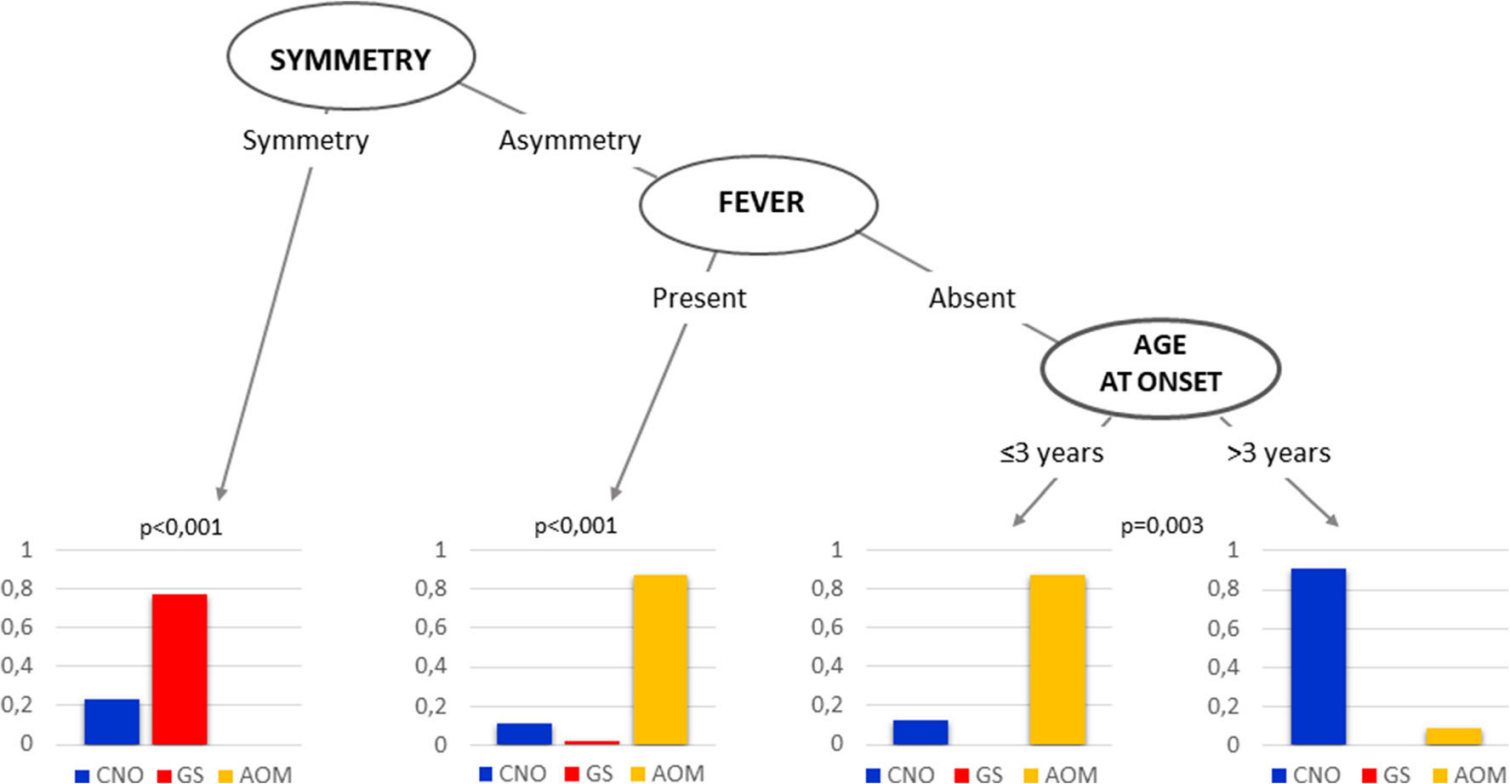
Algorithm for the differential diagnosis in patients with juvenile osteoperiostitis. AOM acute osteomyelitis, CNO chronic non-bacterial osteomyelitis, GS Goldbloom syndrome

From the initial 30 variables considered, after performing a bivariate analysis, a set of nine covariates entered into the R’s *Classification Tree* procedure. This program finally selected three variables, able to significantly differentiate the three diagnostic groups, AOM, CNO and GS, from each other (Fig. 1). The first decisional node of the algorithm was the symmetry of bone involvement (Fig. 2). Symmetrical localization allows one to exclude AOM and to consider, with different probability, the other two conditions: 80% probability for GS, 20% for CNO. In the presence of asymmetrical bone involvement and fever, the probability of AOM is very high. In the absence of fever, the diagnosis of AOM in patients aged ≤ 3 years and of CNO in older children was more likely, respectively. The level of performance of the classification algorithm, carried out by comparing the diagnoses provided by the model and the real diagnoses, showed 85.9% accuracy.

## Discussion

Up to now, there are no gold standards to easily differentiate JOP from each other. In fact, the demonstration of a specific pathogen is reported in only 30–50% of patients with AOM [1], biopsy is useful only to differentiate osteitis from malignancy [10] and, unfortunately, radiological imaging is hardly able to differentiate infectious from non-infectious processes [11]. As for biological markers, the common acute phase reactants, ESR, CRP and even serum procalcitonin have shown low sensitivity and specificity in differentiating these conditions [12].

Furthermore, patients with JOP are followed by various specialists such as general pediatricians, rheumatologists, and orthopedic and infectious disease specialists whose diagnostic approach is often diverse according with the specific cultural and professional background.

Since the diagnosis of the various types of JOP is often difficult and delayed, we thought to be important to early differentiate these entities in order to avoid invasive diagnostic investigations and inappropriate treatments.

In this study, we analyzed the features of these three conditions at disease onset and compared them in order to find peculiar aspects that may help in the differential diagnosis.

Thanks to an innovative statistical procedure, the *R’s Classification Tree* program, among thirty initial variables, nine have shown significance in differentiating the three diseases by bivariate analysis. Eventually, the *R’s Classification Tree* program selected only three variables representing the main nodes of the final classification algorithm: symmetry of bone involvement, presence of fever and age at onset. The level of performance of this classification algorithm, carried out by comparing the diagnoses provided by the model and the real diagnoses, showed a high degree of accuracy (85.9%). Therefore, it allows addressing the diagnostic work-up with high probability of rapidly leading to the correct diagnosis.

We acknowledge some limits of our study that was retrospective and heterogeneous in that patients were drawn from different sources. However, the method of data extraction was uniform, and cases with incomplete data were excluded from the analysis. In addition, the initial radiological imaging of the patients came not only from our Center but also from the peripheral hospitals where some of them were admitted in the first days of their illness. This has certainly reduced the level of the procedure standardization. However, the subsequent independent analysis of the images by two radiologists, specialized in pediatric musculoskeletal diseases, has allowed minimizing any possible technical bias.

In conclusion, we propose a clinical diagnostic algorithm that may help general pediatricians and other physicians taking care of a child with suspicious JOP, in the differential diagnosis among various conditions with high level of accuracy. A prospective validation of this tool, in a wider cohort of patients, is warranted.

## Data Availability

The dataset used during the current study is available from the corresponding author on reasonable request.

## Abbreviations

CRP: C-reactive protein
ESR: Erythrocyte sedimentation rate
MRI: Magnetic resonance imaging
PLT: Platelet count
WBC: White blood cell count
